# Health-related quality of life in patients with alcohol use disorder: comparing instruments and mapping from clinical measures to preference-based measures

**DOI:** 10.1007/s10198-025-01816-0

**Published:** 2025-07-24

**Authors:** Eva Rodríguez-Míguez, Jacinto Mosquera

**Affiliations:** 1 Departamento de Economía Aplicada, ECOBAS, Universidade de Vigo, GRiEE, 36310 Vigo, España; 2 Instituto de Investigación Sanitaria Galicia Sur, Hospital Álvaro Cunqueiro, 36213 Vigo, España; 3 Galician Health Service (SERGAS), Vigo, Spain

**Keywords:** Alcohol use disorder, HRQoL, EQ-5D, SF-6D, AlcQ-4D, AUDIT, DSM-5, Mapping

## Abstract

**Background and objectives:**

The impact of alcohol use disorder (AUD) on quality of life can be quantified by generic and specific measures. This study’s aims are to compare different preference-based instruments to measure health-related quality of life (HRQoL) in patients with AUD and to examine their relationship with alcohol-specific measures used in the clinical setting.

**Methods:**

A sample of 259 patients with AUD were recruited from a Spanish alcoholism unit. We administered EuroQoL–5Dimension –5levels (EQ-5D), ShortForm–6Dimension (SF-6D), AlcoholQuality-of-life–4Dimension (AlcQ-4D), Diagnostic and Statistical Manual of Mental Disorders (DSM-5), and Alcohol Use Disorders Identification Test (AUDIT) instruments at the beginning of treatment and 12 months thereafter. Differences in HRQoL instruments scores were estimated and their capacity to discriminate among known clinical severity groups was analysed. Several mapping functions were tested to transform scores from the clinical setting (AUDIT or DSM-5) into HRQoL scores (EQ-5D, SF-6D or AlcQ-4D).

**Results:**

HRQoL scores are sensitive to the instrument used. Mean scores are always highest using EQ-5D, followed by SF-6D and AlcQ-4D. All HRQoL instruments discriminate among clinical severity groups defined using DSM-5 or AUDIT. Although several mapping functions were estimated, those using the total score of the clinical instruments were selected.

**Conclusion:**

The results suggest that clinical measures used in the field of AUD could be adapted for use in economic evaluation. However, the incremental cost–utility ratio of AUD programs, and hence the policy decisions derived from it, may depend on the HRQoL instrument used.

## Introduction

Alcohol consumption is a serious public health problem in large parts of the world. Alcohol is consumed by more than half of the population in three World Health Organization (WHO) regions: the Americas, Europe, and the Western Pacific. Alcohol use has been linked to more than 200 health conditions ranging from liver diseases to cancers, cardiovascular diseases, suicides, tuberculosis, and HIV/AIDS, among others. Of course, these conditions have a major impact on both mortality and morbidity. It is estimated that alcohol abuse causes 4.7% of deaths worldwide, mostly in the young population; for those between 20 and 39 years old, 13% of all deaths are attributable to alcohol. With regard to morbidity, the risk factor of alcohol use contributes 4.6% to the global burden of disease and injury as calculated in terms of disability-adjusted life years (DALYs); it is the ninth leading risk factor globally and the leading risk factor among males aged 15–49 years [1]. This state of affairs poses a huge problem at both the individual and the societal level. Therefore, public health priorities should include the early detection and diagnosis of alcohol use disorder (AUD), the comprehensive assessment of its consequences, and the cost-effectiveness analysis of treatment options.

In the clinical setting, problems related to alcohol use are assessed by clinical interviews and by means of questionnaires and widely used diagnostic criteria. The most popular questionnaire is the Alcohol Use Disorders Identification Test (AUDIT) proposed by the WHO [2] and validated in Spanish [3]. This questionnaire enables the detection of risky consumption and alcohol use disorder by means of 10 questions. The diagnosis of AUD usually references criteria of the American Psychiatric Association, whose Diagnostic and Statistical Manual of Mental Disorders DSM-5 [4] lists 11 criteria; a diagnosis of AUD is indicated by the presence of at least two criteria for a duration of at least 12 months. A recent revision of this manual (DSM-5 TR) [5] did not introduce any changes in this diagnostic category.

These instruments, of unquestionable usefulness for clinical management, have a crucial limitation in that they do not incorporate a measurable approximation of the consequences of AUD on health-related quality of life (HRQoL). Several instruments, both generic and specific, are available to measure HRQoL in relation to drinking behaviour, alcohol use disorder, and treatment outcomes [6–8]. The most widely used generic instruments are the Short Form 36 (SF-36) – or its reduced versions, such as the Short Form 12 (SF-12) or the Short Form–6 Dimension (SF-6D) [9–20] – and either the 3- or 5-level version of the EuroQoL–5 Dimension (EQ-5D) [21–25]. The cited studies support the suitability of these instruments for measuring not only how alcohol dependence affects HRQoL but also the effects of different treatment interventions. That said, other studies describe the limitations of these instruments in terms of their capacity to discriminate adequately between the health states of patients with alcohol dependence [26, 27] or to measure treatment effects [7, 28]. Within the field of alcohol consumption, there are also specific instruments to measure HRQOL; these include the AQoLS [29], the AQoL9 [30], and one proposed by Rodríguez-Míguez and Mosquera [31] ––hereinafter referred to as ALCohol Quality-of-life–4 Dimension (AlcQ-4D). One advantage of these instruments is that they enable the analysis of quality-of-life dimensions that are strongly affected by AUD but that are not addressed by generic instruments (family relationships, social life, etc.).

However, not all of the HRQoL instruments mentioned produce scores with the necessary properties for deriving quality-adjusted life years (QALYs) [32]. QALYs are the outcome measure used in cost-utility analyses (CUAs), which are the most frequently recommended type of economic evaluation by health technology assessment agencies to inform decisions about the allocation of healthcare resources [33]. In order to calculate QALYs, it is essential that HRQoL instruments produce preference-based cardinal weights, also referred to as preference scores, utility scores, or simply utilities. The SF-6D and EQ-5D are the most widely used generic preference-based instruments for estimating utility scores. To the best of our knowledge, the AlcQ-4D is the only AUD-specific instrument that enables the estimation of utility scores. However, such instruments are not routinely used in clinical settings; instead, risk detection or diagnostic tools such as the AUDIT or DSM-5 are commonly employed. This underscores the importance of developing mapping algorithms that can translate clinical scores into utility scores, thereby enabling QALY estimation. While using mapping functions is a suboptimal solution compared to the direct use of a preference-based measure [34, 35], it is becoming more popular as it enables researchers to estimate utility scores using clinical measure data when preference-based instruments are unavailable. As far as we know, in the AUD field there is no study that describes how one would transform the scores from AUDIT or DSM-5 instruments into HRQoL scores measured in utilities. The only exception is a study by Chavez et al. [36], who found no correlation between AUDIT-C (a reduced version of AUDIT) and the EQ-5D instrument.

The general objective of this research is to compare different instruments for measuring HRQoL in utility scores and to examine their relationship with instruments used in the clinical setting for the screening and diagnosis of alcohol use disorder. We use a sample of patients with this pathology and compare two clinical instruments (DSM-5 and AUDIT) and three instruments that allow us to quantify the impact on utilities: two generic instruments (EQ-5D and SF-6D) and one specific to AUD (AlcQ-4D).

More specifically, the study’s objectives are as follows.

1. To analyse whether the HRQoL of patients with AUD differs depending on the instrument (EQ-5D, SF-6D, or AlcQ-4D) used to measure it; we assess differences among these instruments in measuring patients’ quality of life and the gains from a treatment program.
2. To estimate the correlation between scores derived from HRQoL instruments and those obtained via AUDIT and DSM-5, the clinical questionnaires commonly used for the detection and diagnosis of this pathology.
3. To analyse the ability of the HRQoL instruments to discriminate among levels of clinical severity.
4. To estimate the relationship between HRQoL scores and clinical scores using statistical models to analyse the “exchange rates” between instruments.

The paper is organised as follows. Section"Methods"describes the methodology used in this study, including the sample and study design, a description of the clinical and HRQoL instruments used, and the statistical analyses performed. Section"Results"presents the main findings of the study. The HRQoL and clinical instruments are compared, and the mapping functions to transform clinical scores into utility scores are proposed. Section ‘’ Discussion’’ provides a critical review of our findings and presents the main conclusions.

## Methods

### Sample and study design

The sample of 259 patients with AUD was obtained from a 12-month open, non-randomized, prospective study conducted during 2021–2022. The patients were recruited from a standard alcoholism treatment unit within the public health system of Galicia (a region of Spain). The patients met the following criteria: (a) were aged 18 years or more; (b) were attending their first consultation in the unit; (c) had no cognitive impairment that prevented study participation; (d) were diagnosed with AUD; and (e) had signed their informed consent. Sample size was estimated for a 95% confidence interval and 80% statistical power and to recognize as statistically significant a difference of at least 0.04 units. Note that the minimally important mean difference estimated (in the literature) for the SF-6D and EQ-5D is 0.041 and 0.074, respectively [37].

Patients were interviewed at the beginning of treatment and, when possible, 12 months later. The treatment consists of psychosocial interventions, pharmacological interventions, or both, according to an individualized therapeutic plan, which may vary during the analysis period. Although 259 patients completed the questionnaire at the beginning of the study, 57 did not participate in the 12-month interview because they could not be located, died or refused to participate in the follow-up interview.

The questionnaire (conducted by a social worker, psychologist, or doctor) was divided into three parts. The first part covered the patient’s socioeconomic characteristics, consumption profile, level of motivation, use of other drugs, and chronic illnesses. In the second part, participants completed two clinical questionnaires related to alcohol consumption, the AUDIT and the DSM-5. In the third part, respondents completed two generic HRQoL questionnaires, the EQ-5D-5L (the 5-level version of the EQ-5D, hereafter simply the EQ-5D) and the SF-6D, and the AUD-specific questionnaire, AlcQ-4D. The EQ-5D and SF-6D were selected because they are the HRQoL instruments most often used to estimate utilities; AlcQ-4D was chosen because, despite being a specific (non-generic) instrument, it allows for the estimation of utilities and so its scores can be compared with those derived from the generic HRQoL instruments.

### HRQoL and clinical instruments

The EQ-5D is a generic instrument for measuring HRQoL with five dimensions: mobility, self-care, usual activities, pain/discomfort and anxiety/depression. For this study, we will use the five-level version of the EQ-5D questionnaire [38]. Each dimension has five levels: no problems, slight problems, moderate problems, severe problems and extreme problems. The EQ-5D utility scores are derived from a weighting algorithm. These weights are anchored on a scale where 0 represents death and 1 represents perfect health. Negative values indicate health states that are considered worse than death. In our study, the EQ-5D utility scores were obtained by applying the scoring algorithm estimated for the Spanish population [39].

The SF-6D is a generic instrument with six dimensions: physical functioning, role limitations, social functioning, pain, mental health and vitality. Each dimension has several levels of severity, with physical functioning and pain having six levels, and role limitations, social functioning, mental health and vitality, each having five levels. The SF-6D utility scores are derived from the scoring algorithms estimated for the Spanish population [40]. As with the EQ-5D, these weights are anchored on a 0–1 scale (dead-perfect health).

The AlcQ-4D is a specific instrument that measures the consequences of alcohol-related disorders on the quality of life in four dimensions: family, physical health, psychological health and social consequences. Each dimension has three levels of severity according to the impact produced: none or almost none, moderate and severe. The AlcQ-4D utility scores are also derived from the scoring algorithms estimated for the Spanish population [31] and are anchored on a scale of 0–1 (dead-perfect health).

The AUDIT is a screening tool developed to identify people with alcohol use disorders [2, 3]. It consists of ten questions designed to assess the frequency and quantity of alcohol consumption; signs of dependence, such as difficulty to control drinking, prioritizing drinking over other activities and drinking in the morning; and harmful consequences of drinking, including injury and problems with social, occupational or legal responsibilities. Each question is scored from 0 to 4, with higher scores indicating a greater risk of alcohol-related problems. The AUDIT is measured on a 0–40 scale, where 0 indicates that the individual is an abstainer or has no alcohol-related problems (score 0 on all 10 AUDIT dimensions) and 40 corresponds to the worst situation (score 4 on all dimensions). This scale maps to WHO guidelines [41] that posit four levels of severity: abstinent or low risk (0–7), medium level of alcohol problem (‘hazardous’ drinking) (8–15), harmful drinking (16–19), and possible alcohol dependence (20–40).

The DSM-5 is a diagnostic tool used to identify problematic patterns of alcohol use. It consists of eleven questions, including consuming more alcohol than intended, unsuccessful attempts to reduce or control drinking, and spending significant time obtaining, using, or recovering from alcohol. It also includes recurrent alcohol use leading to failure to meet important responsibilities, persistent social or interpersonal problems, and neglect of important activities due to alcohol use, among others. According to the American Psychiatric Association [4], the DSM-5 is measured on a 0–11 scale ––0 indicates no problems in any of the 11 items, and 11 indicates problems in all items –– with three levels of severity: mild (2–3), moderate (4–5) and severe (6–11).

### Data analysis

The first requirement is a descriptive analysis of the sample. Because the measurement of treatment impact relies solely on data from the 202 patients who completed both the initial and follow-up interviews, and the remaining patients are used in subsequent analyses, descriptive statistics are presented separately for each subsample. Furthermore, although the primary aim of this study is to compare instruments rather than evaluate treatment outcomes, comparing the two groups also allows us to assess the potential risk of attrition bias in estimating treatment effects.

The EQ-5D, SF-6D, and AlcQ-4D utility scores were calculated at baseline and also about 12 months later, applying the scoring algorithms estimated for the Spanish population [31, 39, 40]. The weights of the instruments can be compared because all of them quantify the quality of life in utility scores anchored on a 0–1 (dead-perfect health) scale. We also calculated clinical instrument scores and constructed severity intervals. These instruments cannot be compared because they measure different constructs (screening vs. diagnosis) and use different scales.

Parametric and non-parametric tests (the paired sample mean difference test and Wilcoxon signed-rank test) were used to analyse whether the HRQoL of patients with AUD differs depending on the measuring instrument (EQ-5D, SF-6D, or AlcQ-4D). Inter-instrument differences were analysed in both the baseline and 12-month follow-up data sets. We also tested whether the gain, or the difference between the baseline and 12-month follow-up scores, is significantly different between instruments. We remark that if differences between instruments remain constant across the utility distribution, then it should be possible to find differences between instruments at baseline and 12-month follow-up without any differences in the gain. For this analysis, only patients who completed the baseline and follow-up surveys were included (*n* = 202); for the remainder of the analysis, the data pool (*n* = 259) was considered.

Comparing HRQoL scores with clinical scores required different analyses of the data pool (baseline and follow-up). First, we calculated the Pearson and Spearman correlation coefficients between all instruments. Second, we used the mean difference test and the Mann–Whitney U test to assess the discriminatory capacity of our HRQoL instruments to identify severity groups predicted by the clinical instruments.

Finally, to estimate the relationship between HRQoL and clinical instruments, we performed a regression analysis in which the dependent variable was HRQoL scores (EQ-5D, SF-6D, or AlcQ-4D) and the independent variable was different specifications of the clinical instruments. For each HRQoL instrument, we estimated five models that differed in terms of the independent variable: three models using the AUDIT and two models using the DSM-5. In the case of AUDIT, the following specifications were used as independent variables: global score, calculated by summing the scores across all dimensions (a squared term was also tested to explore potential non-linear effects); dimension scores (it is assumed that the scores obtained in each dimension can have a different weight across dimensions, therefore there are as many independent variables as dimensions); and item levels (the weight for each level on each dimension is estimated separately, estimating as many parameters as the number of items in the instrument). Global and dimension scores were treated as continuous variables, and item levels were modeled as discrete variables. In the case of dimension scores and item responses, models were fitted by backward regression removing non-significant dimensions (*p* > 0.05) and grouping two consecutive levels once it has been determined both that their coefficients are not significantly different from each other and that the signs are inconsistent. To control for multiple testing, p-values were evaluated using the Bonferroni correction. The same strategy was adopted with the DSM-5, although only two models were estimated, since using either dimension scores or item levels yields the same model **—**global scores were treated as continuous variables and dimensions scores as discrete variables.

A random-effects regression was used in all models, given that most patients contributed to two sets of observations (at baseline and at the 12-month follow-up), resulting in non-independent data. We believe that it is appropriate to work with the entire data pool because the more severe states are prevalent at baseline whereas the milder states are prevalent in follow-up data. So, if our transformation function is to cover the broad spectrum of possible situations, it will be especially useful to work with all of the data pool. For each model, we also report the estimated R^2^ values, the mean absolute error (MAE), the root mean square error (RMSE) and the Akaike information criterion (AIC).

In the absence of an external dataset, this study adopts an internal validation strategy based on the hold-out method [34]. The full dataset was randomly divided into two non-overlapping subsets using Stata’s random number generator: 75% of the observations were allocated to the estimation sample, and the remaining 25% were used for validation purposes [42]. This strategy aims to enhance generalizability and reduce the risk of overfitting. Following standard practices in predictive modelling, model performance was evaluated by comparing predicted and observed utility values using the RMSE and MAE on the validation sample, rather than on the estimation sample. Model selection was based on the lowest RMSE and MAE values, taking into account whether the differences in these metrics were statistically significant, as assessed by using both the paired t-test and the Wilcoxon signed-rank test.

This study was approved by the Committee on Ethics of Clinical Research in Galicia (reference code 2017/177). Signed informed consent was obtained from all participants in the study before enrollment. Statistical analyses were performed using Stata software.

## Results

### Sample description

Table 1 presents the sample’s main characteristics at baseline and distinguishes between participants who completed the follow-up interview (the “traced” sample) and those who dropped out of the study. With regard to possible attrition bias, the traced sample and the dropout sample exhibit no significant differences in socioeconomic characteristics. Results from the HRQoL and clinical instruments suggest lower severity profiles in the dropout sample, although the AlcQ-4D and AUDIT scores do not show significant differences.

**Table 1:** Baseline sample description: traced sample vs. drop-out sample

		Traced (*n* = 202)	Drop-out (*n* = 57)	*p-value*^*1*^
EQ-5D score (mean)		0.727	0.800	***0.010***
SF-6D score (mean)		0.558	0.666	***0.004***
AlcQ-4D score (mean)		0.504	0.552	*0.133*
AUDIT score (mean)		21.990	22.632	*0.564*
DSM score (mean)		7.233	6.509	***0.036***
Gender (% men)		73.27	80.7	*0.253*
Age (mean)		50.322	47.667	*0.095*
Education (%)	Less than primary	3.64	8.91	*0.290*
	Primary	47.27	52.97	
	Secondary	36.36	25.25	
	University	12.73	12.87	
Type of family living (%)	Alone	26.73	21.05	*0.537*
	With a partner	35.64	36.84	
	With family of origin (no with a partner)	15.84	12.28	
	Other	21.78	29.82	
Labor status (%)	Employed	31.19	38.6	*0.208*
	Unemployed	33.66	38.6	
	Inactive	35.15	22.81	
Standard beverage units per week		55.22	70.35	***0.043***
Dominant consumption profile (%)	Daily	78.95	66.83	*0.214*
	Weekend	5.26	8.42	
	Mixed	15.79	24.75	
Dominant type of beverage (%)	Wine	27.36	10.53	***0.010***
	Beer	29.85	50.88	
	Liquor	10.45	10.53	
	Mixed	32.34	28.07	
Level of motivation (%)	Precontemplation	5.5	3.51	*0.282*
	Contemplation	30	22.81	
	Preparation	31	26.32	
	Action	33.5	47.37	
Other drug use (%)	Tobacco	62.38	66.67	*0.553*
	Hashish	11.39	17.54	*0.218*
	Cocaine	8.42	14.04	*0.205*
	Other	0.99	3.51	*0.173*

^*1*^ Differences between samples tests

*EQ-5D*: EuroQoL 5 Dimension, *SF-6D*: Short Form 6 Dimension*, AlcQ-4D*: Alcohol Quality-of-life 4 Dimension, *DSM-5*: Diagnostic and Statistical Manual of Mental Disorders, *AUDIT*: Alcohol Use Disorders Identification Test

### Comparing HRQoL instruments

**Fig. 1:**
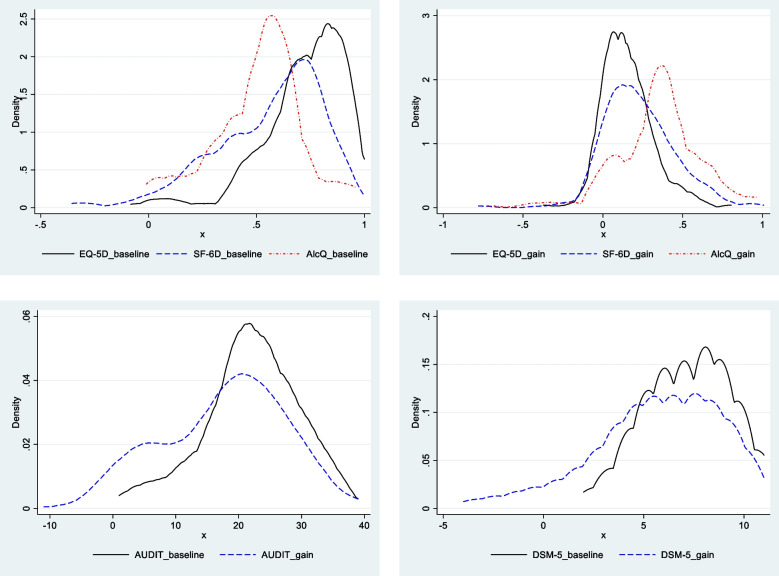
Distribution of baseline scores and treatment impact by instruments. *EQ-5D*: EuroQoL 5 Dimension, *SF-6D*: Short Form 6 Dimension*, AlcQ-4D*: Alcohol Quality-of-life 4 Dimension, *DSM-5*: Diagnostic and Statistical Manual of Mental Disorders, *AUDIT*: Alcohol Use Disorders Identification Test

Table 2 presents the mean scores for all instruments at baseline and at the 12-month follow-up. The estimation of quality of life in patients with AUD is sensitive to the HRQoL instrument used. Parametric and non-parametric tests revealed that, in both analysis periods, there are significant differences (at the 1% level) between all instruments, except between EQ-5D (12-month) and AlcQ-4D (12-month), whose difference is significant at the 5% level (*p* = 0.02) with the paired-sample mean difference test and not significant with the Wilcoxon signed-rank test (*p* = 0.67). The mean score is always significantly lower in SF-6D than in EQ-5D; the AlcQ-4D instrument yields the lowest mean scores at baseline but occupies an intermediate position in the follow-up. Table 2 also shows that treatment has a positive effect on patients’ quality of life and in reducing the severity scores of clinical instruments; all gains are statistically significant (*p* < 0.001). Among the HRQoL instruments, the AlcQ-4D estimates a significantly greater treatment effect, followed by the SF-6D and the EQ-5D. Figure 1 plots, for each HRQoL instrument, the distribution of initial scores and of gains.

**Table 2:** Scores of instruments

	Baseline						12 months						Gain	
	Mean	SD	Median	IQR	Min	Max	Mean	SD	Median	IQR	Min	Max	Mean	SD
EQ-5D	0.73	0.19	0.74	0.23	−0.08	1.00	0.88	0.13	0.92	0.16	0.33	1.00	0.15^**^	0.16
SF-6D	0.56	0.25	0.64	0.30	−0.36	1.00	0.78	0.18	0.84	0.16	−0.10	1.00	0.23^**^	0.23
AlcQ-4D	0.50	0.21	0.57	0.27	−0.01	0.96	0.85	0.19	0.96	0.16	0.09	0.96	0.35^**^	0.26
AUDIT	21.99	7.55	22.00	9.00	1.00	39.00	4.41	7.60	0.00	6.00	0.00	36.00	17.58^**^	9.74
DSM-5	7.23	2.19	7.00	3.00	2.00	11.00	1.52	2.89	0.00	2.00	0.00	11.00	5.71^**^	3.29
*n* = 202														

^**^ Significant at the 1% level (paired sample mean difference test and Wilcoxon signed-rank test were used)

All HRQoL instruments (EQ-5D, SF-6D, and AlcQ-4D) are different from each other at the 1% level, both at baseline and at 12 months *except* EQ-5D-5L (12 months) vs. AlcQ-4D (12 months), which are different at the 5% level (*p* = 0.02) with the paired-sample mean difference test and are not significantly different with the Wilcoxon signed-rank test (*p* = 0.67). AUDIT and DSM-5 are not comparable with each other or with EQ-5D, SF-6D and Alc-4D because they are based on different measurement scales

*EQ-5D*: EuroQoL 5 Dimension, *SF-6D*: Short Form 6 Dimension*, AlcQ-4D*: Alcohol Quality-of-life 4 Dimension, *DSM-5*: Diagnostic and Statistical Manual of Mental Disorders, *AUDIT*: Alcohol Use Disorders Identification Test, *SD* Standard deviation; *IQR* Interquartile range; *Min* Minimum; *Max* Maximum

### Comparing clinical and HRQoL instruments: Discriminant ability of HRQoL instruments

Table 3 reports the correlation coefficients between the different instruments; all values are significant at the 1% level. The highest correlations are between the EQ-5D and SF-6D and between DSM-5 and AUDIT. As for the correlation between quality-of-life and clinical instruments, the AlcQ-4D has the highest correlation (a correlation of more than − 0.7 with both instruments); neither SF-6D nor EQ-5D correlates more than − 0.60 with any of the clinical instruments.

**Table 3:** Correlation between instruments (Spearman rho/Pearson r)

	EQ-5D	SF-6D	AlcQ-4D	AUDIT
SF-6D	0.835/0.837			
AlcQ-4D	0.652/0.654	0.710/0.693		
AUDIT	−0.519/−0.481	−0.580/−0.544	−0.732/−0.716	
DSM-5	−0.531/−0.495	−0.605/−0.562	−0.733/−0.719	0.865/0.878

Observations = 461; *n* = 259

All values are significant at the 1% level

*EQ-5D*: EuroQoL 5 Dimension, *SF-6D*: Short Form 6 Dimension*, AlcQ-4D*: Alcohol Quality-of-life 4 Dimension, *DSM-5* Diagnostic and Statistical Manual of Mental Disorders; *AUDIT* Alcohol Use Disorders Identification Test

Table 4 gives the estimated mean HRQoL for each of the severity groups as derived from the clinical instruments, which allows us to analyse the discriminant ability of HRQoL instruments. All three of them can discriminate among severity levels, but the discriminating capacity of AlcQ-4D and SF-6D is marginally greater: (a) AlcQ-4D finds significant differences at the 1% level between all groups except between AUDIT-level2 and AUDIT-level3, which are different at the 5% level; (b) SF-6D finds significant differences at the 1% level between all groups, except between DSM-level2 and DSM-level3, which are different at the 5% level, and does not discriminate between AUDIT-level3 and AUDIT-level4; (c) EQ-5D finds significant differences at the 1% level between all groups except between AUDIT-level3 and AUDIT-level4, which are different at the 5% level, and does not discriminate between AUDIT-level2 and AUDIT-level3 and between DSM-level2 and DSM-level3. The EQ-5D invariably produces higher scores than the SF-6D in all severity groups, and SF-6D yields higher scores than AlcQ-4D *except* for the mildest levels of AUDIT and DSM-5.

**Table 4:** HRQoL values by clinical severity group (n = 461)

			EQ-5D		SF-6D		AlcQ-4D	
		*n*	Mean	SD	Mean	SD	Mean	SD
AUDIT_level 1 (A1)		169	**0.900**	0.111	**0.819**	0.138	**0.892**	0.142
AUDIT_level 2 (A2)		53	**0.821**	0.157	**0.717**	0.185	**0.666**	0.200
AUDIT_level 3 (A3)		55	**0.785**	0.152	**0.612**	0.228	**0.563**	0.230
AUDIT_level 4 (A4)		184	**0.714**	0.197	**0.540**	0.255	**0.482**	0.208
			*A2 vs A3 (p* = *0.238;0.153)* *A3 vs A4 (p* = *0.014;0.012)*		*A3 vs A4 (p* = *0.061;0.059)*		*A2 vs A3 (p* = *0.015;0.022)*	
DSM-5_level 1 (D1)	185		**0.899**	0.107	**0.818**	0.133	**0.878**	0.152
DSM-5_level 2 (D2)	59		**0.768**	0.198	**0.643**	0.232	**0.609**	0.193
DSM-5_level 3 (D3)	207		**0.731**	0.186	**0.553**	0.249	**0.494**	0.219
			*D2 vs D3 (p* = *0.182;0.070)*		*D2 vs D3 (p* = *0.013;0.010)*			

All differences between severity groups are significant at the 1% level, except where p-values (*p*) are reported. Two p-values are shown: the first corresponds to the mean difference test and the second to the Mann–Whitney U test

*HRQoL*: health-related quality of life, *EQ-5D*: EuroQoL 5 Dimension, *SF-6D*: Short Form 6 Dimension*, AlcQ-4D*: Alcohol Quality-of-life 4 Dimension, *DSM-5*: Diagnostic and Statistical Manual of Mental Disorders, *AUDIT*: Alcohol Use Disorders Identification Test

### Mapping between clinical scores onto HRQoL scores

Table 5 summarizes results from the different regressions estimated. In the case of AUDIT, three models are shown for each HRQoL instrument according as how the dependent variable is defined: as the total score (AUDIT-model 1), as the score of (significant) dimensions (AUDIT-model 2), or as levels of (significant) dimensions (AUDIT-model 3). In the DSM-5 case, two models per instrument are presented due to the binary nature of each dimension: one that considers the total score as the dependent variable (DSM-Model 1), and another that considers the (significant) dimensions (DSM-Model 2). Quadratic terms were also tested in Model 1 but excluded as they were not statistically significant. Table 5 also reports the values for each model’s R^2^, AIC, MAE and RMSE. Although model selection is based on the MAE and RMSE from the validation sample (with results shown below), some general conclusions can be drawn regarding the fit of the estimated models. Typically, the RMSE and MAE values across the different models are very similar for each combination of clinical measure (AUDIT or DSM) and HRQoL instrument (EQ-5D, SF-6D or AlcQ-4D), with differences consistently below 0.01. As expected, models with more predictors tend to yield higher R^2^ values. However, the AIC, which penalises models with more parameters, does not exhibit a clear pattern. For the AUDIT, Model 2 shows the lowest AIC for the SF-6D and AlcQ-4D, and it should not be disregarded for the EQ-5D, given that the difference in AIC between Models 2 and 3 is within the 4–7 range [43]. For the DSM, Model 1 yields the lowest AIC for the SF-6D and AlcQ-4D, while Model 2 has the lowest AIC value for the EQ-5D.

**Table 5:** Relationship between HRQoL scores and clinical scores

	Dependent variable: HRQoL score					
		**EQ-5D**		**SF-6D**		**AlcQ-4D**
	Coef	Std. Err	Coef	Std. Err	Coef	Std. Err
AUDIT-Model 1 (Independent variable: score AUDIT)						
Total score	−0.008^**^	0.001	−0.012^**^	0.001	−0.017^**^	0.001
cons	0.916^**^	0.013	0.840^**^	0.017	0.895^**^	0.016
*R*^*2*^*w/R*^*2*^*b/R*^*2*^*o*	*0.512/0.166/0.245*		*0.558/0.234/0.323*		*0.681/0.405/0.519*	
*RMSE/MAE/AIC*	*0.116/0.116/−307.188*		*0.153/0.147/−145.901*		*0.173/0.137/−181.480*	
AUDIT-Model 2 (Independent variable: score dimensions AUDIT)						
Dim 1	−0.028^**^	0.005	−0.042^**^	0.007	−0.074^**^	0.007
Dim 5	−0.026^**^	0.007	−0.038^**^	0.009	−0.036^**^	0.009
Dim 6	−0.030^**^	0.007	−0.042^**^	0.009	−0.044^**^	0.009
cons	0.920^**^	0.014	0.837^**^	0.017	0.908^**^	0.017
*R*^*2*^*w/R*^*2*^*b/R*^*2*^*o*	*0.563/0.221/0.303*		*0.595/0.295/0.375*		*0.693/0.456/0.547*	
*RMSE/MAE/AIC*	*0.111/0.111/−332.131*		*0.147/0.143/−170.413*		*0.172/0.134/−198.446*	
AUDIT-Model 3 (Independent variable: levels dimensions AUDIT)						
Dim 1 (ref: level 0)						
Level 1	**−0.016**	0.031	**−0.060**	0.041	−0.156^**^	0.034
Level 2	**−0.045**	0.033	**−0.068**	0.043	−0.223^**^	0.037
Level 3	−0.071^**^	0.027	−0.106^**^	0.035		
Level 4	−0.122^**^	0.021	−0.180^**^	0.027	−0.315^**^	0.028
Dim 5 (ref: level 0–1)						
Level 2	**−0.046**	0.025	−0.068^*^	0.033	**−0.060**	0.033
Level 3	−0.051^*^	0.024	−0.118^**^	0.031	−0.085^**^	0.031
Level 4	−0.139^**^	0.032	−0.148^**^	0.042	−0.156^**^	0.042
Dim 6 (ref: level 0–2)						
Level 3	**−0.067**	0.038	−0.104^*^	0.049	−0.118^*^	0.049
Level 4	−0.126^**^	0.032	−0.185^**^	0.041	−0.166^**^	0.041
cons	0.913^**^	0.014	0.839^**^	0.019	0.909^**^	0.019
*R*^*2*^*w/R*^*2*^*b/R*^*2*^*o*	*0.598/0.244/0.336*		*0.617/0.303/0.392*		*0.662/0.462/0.535*	
*RMSE/MAE/AIC*	*0.108/0.109/−337.622*		*0.145/0.141/−168.151*		*0.182/0.137/−185.029*	
DSM-Model 1 (Independent variable: score DSM-5)						
Total score	−0.024^**^	0.002	−0.036^**^	0.003	−0.050^**^	0.003
cons	0.913^**^	0.013	0.838^**^	0.017	0.890^**^	0.016
*R*^*2*^*w/R*^*2*^*b/R*^*2*^*o*	*0.485/0.178/0.250*		*0.489/0.296/0.338*		*0.661/0.414/0.512*	
*RMSE/MAE/AIC*	*0.119/0.115/−305.492*		*0.166/0.144/−146.478*		*0.179/0.141/−176.678*	
DSM-Model 2 (Independent variable: dimensions DSM-5)						
Dim 1					−0.094^**^	0.032
Dim 4	−0.065^**^	0.021	−0.076^**^	0.028	−0.076^**^	0.027
Dim 6					−0.143^**^	0.031
Dim 7	−0.062^**^	0.020	−0.118^**^	0.027	−0.093^**^	0.026
Dim 9	−0.056^**^	0.022	−0.101^**^	0.029		
Dim 11	−0.117^**^	0.023	−0.115^**^	0.031	−0.149^**^	0.030
cons	0.907^**^	0.013	0.825^**^	0.017	0.882^**^	0.016
*R*^*2*^*w/R*^*2*^*b/R*^*2*^*o*	*0.539/0.209/0.287*		*0.487/0.307/0.348*		*0.680/0.405/0.520*	
*RMSE/MAE/AIC*	*0.114/0.114/−320.115*		*0.166/0.144/−144.902*		*0.174/0.140/−174.064*	

Observations = 346; **p < 0.01, * *p* < 0.05

*HRQoL*: health-related quality of life, *EQ-5D*: EuroQoL 5 Dimension, *SF-6D*: Short Form 6 Dimension*, AlcQ-4D*: Alcohol Quality-of-life 4 Dimension, *DSM-5*: Diagnostic and Statistical Manual of Mental Disorders, *AUDIT*: Alcohol Use Disorders Identification Test, *R*^*2*^*w*: R^2^ within, *R*^*2*^*b*: R^2^ between, *R*^*2*^*o*: R^2^ overall, *RMSE*: root mean square error, *MAE*: mean absolute error, *AIC*: Akaike information criterion

Table 6 presents the RMSE and MAE obtained by comparing the actual and predicted values in the *validation* sample. For the AUDIT, there are no statistically significant differences between Models 1, 2, and 3 in terms of both MAE and RMSE, regardless of the HRQoL instrument used as a predictor. This conclusion holds both when the paired t-test or the Wilcoxon signed-rank test is used. For the DSM, no significant differences are observed among the models tested for the EQ-5D and SF-6D instruments. However, Model 1 shows statistically lower MAE and RMSE values when using the AlcQ-4D instrument **—** with *p* < 0.01 for both metrics, except for the non-parametric test of RMSE, where *p* = 0.079. In light of these considerations and for the sake of parsimony, we recommend using Model 1, for all combinations of clinical and HRQoL instruments used in the mapping process, along with the corresponding parameters in each case. For example, when it is necessary to convert AUDIT scores into EQ-5D utilities, we propose to use the following mapping function: EQ-5D (estimated) = 0.916–0.008 × AUDIT score. It is worth noting, however, that it is not necessary to have information on all AUDIT or DSM dimensions to achieve a comparable model fit.

**Table 6:** Goodness-of-fit results from validation analysis

		EQ-5D		SF-6D		AlcQ-4D	
		RMSE	MAE	RMSE	MAE	RMSE	MAE
AUDIT	Model 1	0.145	0.116	0.212	0.163	0.183	0.144
	Model 2	0.144	0.113	0.211	0.159	0.189	0.143
	Model 3	0.147	0.114	0.211	0.156	0.191	0.145
DSM-5	Model 1	0.140	0.113	0.206	0.158	0.174	0.133
	Model 2	0.147	0.118	0.219	0.167	0.189	0.149

Observations = 115

*HRQoL*: health-related quality of life, *EQ-5D*: EuroQoL 5 Dimension, *SF-6D*: Short Form 6 Dimension*, AlcQ-4D*: Alcohol Quality-of-life 4 Dimension, *DSM-5*: Diagnostic and Statistical Manual of Mental Disorders, *AUDIT*: Alcohol Use Disorders Identification Test, *RMSE*: root mean square error, *MAE*: mean absolute error

## Discussion

This study aimed to compare different preference-based instruments for measuring HRQoL in patients AUD, and to examine their relationship with alcohol-specific measures commonly used in clinical practice. Regarding the first objective, this study shows that the estimation of HRQoL is sensitive to the instrument used: EQ-5D always produces the highest mean utility score, and SF-6D produces a higher score than AlcQ-4D at baseline but yields the lowest mean scores in the follow-up sample. In addition, HRQoL instruments detect a positive and significant effect of treatment that is also clinically meaningful —the gains estimated using the EQ-5D and SF-6D (0.15 and 0.23, respectively) more than double the minimally important differences reported in the literature [37, 44]. Regarding the second objective although all HRQoL instruments can discriminate between severity levels previously established by clinical instruments, the discriminatory ability of SF-6D and AlcQ-4D is slightly higher than that of EQ-5D. This paper also proposes algorithms that enable using the total scores provided by clinical instruments (AUDIT and DSM) to “predict” preference-based HRQoL scores (EQ-5D, SF-6D, and AlcQ-4D).

One advantage of our study is its use of a sample of patients diagnosed with AUD. The sample is important because general population studies of the relationship between alcohol abuse and HRQoL report conflicting results: some studies find a negative effect on HRQoL [45–47], but other studies report little or no effect [48–53] or even a positive effect [36, 54, 55]. These disparities may be explained, in part, by the difficulty that population-based surveys have in discriminating between AUD and “mere” heavy drinking. In addition, failure of cross-sectional cost studies to identify the long-term effects of this pathology may mask its impact on quality of life; for example, a severe reduction in health that results from heavy drinking may lead to reduced alcohol intake, which might (misleadingly) suggest a positive relationship between low alcohol consumption and poorer quality of life. However, in samples consisting of diagnosed patients (as in our study), the relationship between severity of AUD and poor HRQoL is clear; see the studies reviewed by Ugochukwu et al. [56] or subsequent research [13, 14, 20, 57–60].

Although our study finds a significant negative relationship between AUD severity and HRQoL for all instruments analysed, the results suggest that EQ-5D is less sensitive than the other instruments. Other studies have likewise found that the EQ-5D has some limitations for capturing those quality-of-life dimensions most affected by AUD [7]. At least two causes for this lower sensitivity are worth mentioning. On the one hand, the EQ-5D’s descriptive system focuses mainly on physical condition, and – as Miller and Miller [61] point out – “the dominance of a health approach for addiction treatment may not be reflective of an addict’s main concerns”. It follows that emotional problems, lack of energy and vitality, and the impact of this pathology on social and family life may not be adequately captured by the EQ-5D, which could explain the greater sensitivity of the SF-6D and the AlcQ-4D found in our research. On the other hand, the EQ-5D’s relatively low sensitivity may be due to the “ceiling” effect that characterizes this instrument (i.e., its poor discrimination among mild health states). One of the goals of the 5-level version of the EQ-5D goals was to smooth the ceiling effect of the 3-levels version, which is found also in the alcohol domain [26, 62]. However, there is evidence that the ceiling effect persists (albeit smoothed) [63]. Furthermore, direct comparisons between the 5-level version of the EQ-5D and the SF-6D reveal that the former has a higher ceiling effect than the latter; this difference has been demonstrated both in patient studies [64–66] and in general population studies (e.g., [67]).

With regard to clinical instruments, we have not been able to locate any studies that compare AUDIT and DSM-5 in patients diagnosed with AUD. In population-based studies, there is evidence showing a strong association between AUDIT and DSM-5 for detecting AUD [68, 69]. There is also evidence in favour of AUDIT (as compared with DSM-5) for detecting alcohol misuse among young people [70]. With regard to evaluating model performance, Brazier et al. [34] argue that MAE and RMSE are more appropriate indicators of predictive accuracy than R^2^. The RMSE and MAE of our models ranged from 0.11 to 0.18 and from 0.11 to 0.15, respectively. These values are in line with those reported by Brazier et al. [34] in their systematic review, where the RMSE values ranged from 0.08 to 0.2 and the MAE values from 0.01 to 0.19. The average MAE across all estimated models remains almost the same between the estimation and validation samples (0.132 vs. 0.139). However, the average RMSE, which is more sensitive to outliers, increases from 0.148 to 0.18 between the estimation and validation samples.

This is the first study to propose mapping functions that transform clinical scores into preference-based HRQoL scores within the AUD field. Although Chavez et al. [36] considered two potential mappings –from AUDIT-C scores to EQ-5D and SF-6D utilities– they did not propose any functions as there were no meaningful differences in HRQoL across levels of alcohol consumption. The authors suggest that generic HRQOL measures may not adequately capture differences associated with alcohol use, a very different result from what we obtained. These results might be explained by that study's use of a general population sample, its failure to identify sample members who were diagnosed with AUD (since AUDIT is based on self-reported alcohol consumption), and because people who seek treatment for an AUD tend to be more concerned about their drinking and the consequences of their alcohol abuse.

Our study has several limitations, most of which are related to its external validity. First, the sample size is small. Small samples are common when the population under study consists of patients diagnosed with alcohol problems; in Luquiens et al.’s review [7], less than a third of such studies featured sample sizes larger than 225 participants. Nonetheless, the small sample size here could call into question the generalizability of our results to the wider AUD patient population and may also have affected whether some of the estimated parameters were (or were not) statistically significant. Second, although the population was selected by systematically recruiting the population attending a drug treatment centre, it may not be representative of the profiles of alcohol-dependent persons in other areas. Third, while the use of separate samples for estimation and validation purposes is widely regarded as a more robust approach than assessing predictive performance based solely on model fit, it still does not fully evaluate the model’s performance on truly independent datasets. Finally, internal validity may also be compromised in the absence of a control group, which makes it practically impossible to determine with certainty whether changes in scores on the instruments analysed are due to the treatment intervention or to another confounding variable. Note that intervention outcomes were established by comparing the values of the instruments at the beginning of the treatment and one year later (a control group was not established because this is the usual treatment at the centre and no alternative approach was available for comparison). This limitation would be of considerable relevance if the primary objective of our study had been to measure treatment efficacy. However, it is of minor importance given that our main focus was to compare within-sample differences between instruments.

This study enables us to draw some conclusions relevant for public decision making. First, our results establish that all the HRQoL instruments used allow for discriminating between the AUD condition’s levels of severity and that any of them is a reasonable choice in the AUD setting. Second, we have observed that the HRQoL associated with alcohol use disorders and the effects of treatment intended to manage them are sensitive to the choice of HRQoL instrument. Therefore, the results of economic evaluations can differ depending on which instrument is used. As there is no “gold standard” against which instruments can be compared, no particular approach can be considered superior. In any case, our results anticipate that the incremental cost–utility ratio will be lower – making it more likely that such treatment will receive public funding – when using the SF-6D or the AlcQ-4D than when using the EQ-5D. Third, our findings suggest that failing to incorporate family and social dimensions will lead to underestimating the treatment effect on quality of life. How best to incorporate these variables in the assessment of treatments is still an open question. Finally, given that clinical instruments do not have the psychometric properties necessary for their incorporation into economic evaluation studies, this study also proposes functions that can be used to transform clinical instrument scores into utility scores. In addition, our proposed adjustment functions for each clinical instrument (i.e., one for each HRQoL instrument evaluated) facilitates sensitivity analyses when assessing quality of life and the effect of treatments on patients with alcohol use disorder. Mapping functions are never preferable to using a preference-based measure directly, but they do allow data obtained in the clinical setting to be transformed for use in cost-effectiveness analyses and thereby improve evidence-informed decision making in health policy.

## Data Availability

Data will be available upon reasonable request by contacting the corresponding author.
